# When do supervisors punish subordinates’ unethical pro-organizational behavior: Roles of moral identity and goal congruence with the group

**DOI:** 10.3389/fpsyg.2023.1121317

**Published:** 2023-03-20

**Authors:** Feng Gao, Yao Wang, Jiaojiao Zhang

**Affiliations:** ^1^School of Business, Renmin University of China, Beijing, China; ^2^School of Business, China University of Political Science and Law, Beijing, China

**Keywords:** unethical pro-organizational behavior, moral identity, goal congruence with the group, trust, abusive supervisor behavior

## Abstract

Given that unethical pro-organizational behavior (UPB) violates moral standards but benefits the organization at the same time, supervisors’ responses to this behavior could be equivocal although it is supposed to be punished. Previous research, however, has centered on antecedents of UPB, less is known about its consequences, especially how supervisors respond to subordinates’ UPB. Integrating social identity theory with social information processing theory, this paper aims to explain when supervisors perceive subordinate UPB in a negative way, and further engage in negative leading behaviors as punishments for UPB. Results of a multi-wave, multiple-source survey suggest that subordinates’ UPB is most negatively related to supervisors’ trust when supervisors’ moral identity is prominent and goal congruence with the group is low. Furthermore, results show that reduced trust ultimately elicits abusive supervisor behavior. These findings extend understanding of when and why supervisors punish rather than indulge subordinates who act in ethically questionable ways and provide important insights into supervisors’ leading behavior from a bottom-up perspective.

## Introduction

Although unethical behavior is believed to serve self-interest or harm the organization (e.g., Skarlicki and Folger, 1997; Greenberg, 2002), recent research has suggested that employee’s unethical behaviors could be driven by an organization-serving motivation (Umphress et al., 2010), which is known as *unethical pro-organizational behaviors (UPB)*. Defined as “unethical behaviors conducted by employees to potentially benefit the organization” (Umphress et al., 2010; Umphress and Bingham, 2011), UPB is paradoxical in nature, being unethical while potentially beneficial to the organization in the short term, which challenges supervisors to properly deal with employees who engage in UPB.

However, existing literature did not answer the question of how supervisors respond to employees’ UPB. Indeed, most of the research that explored UPB’s consequences has focused on its impact on employees’ own behavior (e.g., Chen et al., 2022; Jiang et al., 2022) or on peer’s response (e.g., Tang et al., 2021; Zeng et al., 2022), less is known about how supervisors respond to it. This is a critical gap in UPB literature for at least two reasons: First, supervisors are supposed to constrain or even punish those who engage in UPB, in that while UPB is intended to benefit the organization, the final result of this unethical conduct could deviate from their intentions, and even cause destructive outcomes (Umphress et al., 2010; Yan et al., 2021). Second, given that supervisors typically occupy an important position within the organization, they should feel more responsible than subordinates for the organizational interest, and thus are facing a greater dilemma (i.e., organization interest vs. moral principle) to deal with UPB. Taken together, while UPB is supposed to be punished, supervisors’ actual response to it could be equivocal, and relevant research is still lacking, which urges scholars to fill this gap.

Based on this research gap, we aim to explore in this paper when supervisors, as they are supposed to, negatively perceive and punish subordinates’ UPB. Integrating social identity theory (Tajfel and Turner, 1986) with social information theory (Salancik and Pfeffer, 1978), we contend that subordinate UPB implicates contradictory information (i.e., unethical vs. pro-organizational), and how this information is processed by supervisors depends on the processing schema implicit in supervisors’ identities. Specifically, we focus on the contingent role of moral identity and goal congruence with the group, proposing that when supervisors’ moral identity is high and goal congruence with the group is low, they appear most attentive to the immoral aspects while least concerned with the group-serving aspects in UPB. As a result, these supervisors are most likely to view UPB as a lack of adherence to acceptable principles, judging it as unreliable (Fehr et al., 2020), and thus losing trust in the subordinates (McAllister, 1995).

**Figure 1:**
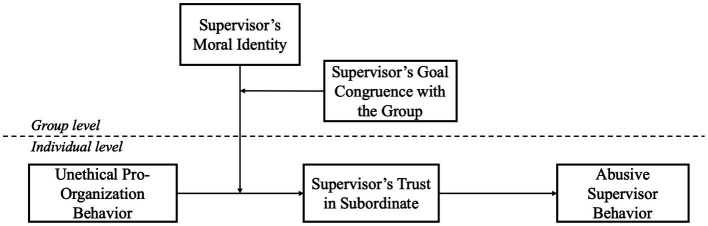
The hypothesized model.

Furthermore, we theorize that subordinate UPB will lead to punitive supervisor behavioral responses from the supervisor. Abusive supervisor behavior, although undesirable, is commonly adopted as an instrument to punish misbehaviors and stimulate performance promotions (Ferris et al., 2007; Tepper, 2007; Krasikova et al., 2013). Thus, we further examine the downstream effect of subordinate UPB on abusive supervisor behaviors. This is important because it reveals when supervisors take actions to prevent and constrain UPB within organizations. Taken together, we posit that supervisors are most likely to conduct abusive behaviors as sanctions to subordinate UPB when their moral identity is high and goal congruence with the group is low. Shown in Figure 1 is our theoretical model.

Our work contributes to the existing literature in several ways. First, by focusing on the impact of UPB on supervisor attitudinal and behavioral consequences, our work complements existing UPB research with supervisors’ responses to UPB, which is an important yet rarely studied perspective. In addition, by integrating social identity theory with social information processing theory, our work also provides a lens to understand the contingencies of supervisors’ decisions on UPB. Second, we go further to examine the indirect effect of subordinates’ UPB on abusive supervisor behavior, which allows us to augment scholars’ understanding of the dyadic interaction between supervisors and subordinates from an upward influence perspective. In addition, given that research on abusive supervision mainly focus on subordinate static traits or characteristics as its antecedents (for reviews, see Zhang and Bednall, 2016; Fischer et al., 2021), this work also adds to our knowledge about its formation as a result of subordinate workplace behaviors (i.e., UPB). Finally, whereas moral identity has been widely studied as a predictor of an individual’s own ethical behaviors (Jennings et al., 2014; Hertz and Krettenauer, 2016), we instead explore its influence on one’s perception of others’ ethic-related actions. Integrating social identity theory with social information theory, the present research enriches our understanding of the role that one’s identities play in the procedure of information processing.

## Theory and hypotheses

### Subordinate UPB and trust in the subordinate

Social information processing theory suggests that individuals rely on surrounding social cues to construct and interpret events, which further affects their expression of attitudes (Salancik and Pfeffer, 1978). In the dyadic interaction between supervisor and subordinate, subordinates’ behaviors can be important social cues for supervisors to derive personal perceptions, attitudes, and actions toward the subordinate. Accordingly, we posit that UPB conveys information and signals that affect supervisors’ trust in their subordinates. Trust, as a willingness to be vulnerable to another party’s actions (Mayer et al., 1995; Colquitt et al., 2007), implicates one’s personal judgments of other’s reliability (McAllister, 1995), which has been referred to as an important attitudinal consequence of both (un)ethical behaviors (e.g., Kennedy and Schweitzer, 2018; Fehr et al., 2020) and pro-organizational behaviors (e.g., Brower et al., 2009) in previous research.

Subordinate UPB, however, implicates contradictory information to supervisors, including both positive (i.e., pro-organizational) and negative (i.e., unethical) cues, which results in paradoxical perceptions of and reactions to it (Wen et al., 2020). On the one hand, people tend to lose trust in those who engage in unethical behaviors, believing that they are unreliable because of their questionable standards and principles (Norman et al., 2010; Ng and Feldman, 2015; Fehr et al., 2020). UPB, given its unethical nature, is considered illegal or morally unacceptable to the larger society (Umphress and Bingham, 2011), thus conveying negative information to the supervisor and decreasing the trust of the supervisor. In a same line, research has identified integrity as a critical indicator of trust development (e.g., Mayer et al., 1995; Simons, 2002; Tomlinson et al., 2020), which suggests that adherence to a set of acceptable principles instills trust, while unethical behaviors go against this rule.

On the other hand, UPB is pro-organizational, intending to benefit the organization or its members (Umphress et al., 2010; Umphress and Bingham, 2011). Research has suggested that pro-organizational behavior could be positively evaluated as an altruistic orientation toward the group and the supervisor, thus being regarded as a signal for trustworthiness (Brower et al., 2009; Knoll and Gill, 2011; Tomlinson et al., 2020). In this way, subordinates who engage in UPB convey positive information to the supervisor, showing that they are concerned about the organizational benefit, thus gaining more trust from the supervisor.

The question then becomes when supervisors will process the contradictory information indicated by UPB more negatively and lower their trust in the subordinates who engage in UPB. According to social identity theory (Tajfel and Turner, 1986), the way individuals process social information could be influenced by their identities. Social identity, as a part of an individual’s self-concept, determines the way one thinks, feels, and behaves in different situations and offers a schema for social information processing (Tajfel and Turner, 1986; Hogg et al., 1995). Specific to the context of UPB, the information processing schema should pertain to both moral identity and organizational identity. Thus, integrating social identity theory with social information processing theory, we propose that the information processing for UPB is contingent on moral identity and goal congruence with the group (i.e., representation of the organizational identity). In the below, we discuss how these contingencies play a role in supervisors’ trust in subordinates who engage in UPB.

#### The role of supervisor moral identity

Having its root in social identity theory (Tajfel and Turner, 1986; Ashforth and Mael, 1989), moral identity is defined as an individual’s “self-conception organized around a set of moral traits,” which describes the importance of morality in one’s sense of self (Aquino and Reed, 2002; Reed and Aquino, 2003). Based on a social identity perspective, moral identity provides individuals with moral schemas that are easily activated for processing social information(Lapsley and Lasky, 2001; Weaver, 2006). Specifically, high-moral identity individuals are more attentive to ethical facets in their environments(Sparks and Hunt, 1998; Daniels et al., 2011), thus focusing more on the moral cues included in other’s actions to shape their cognition and attitudes (Aquino et al., 2007; Wang et al., 2019).

Accordingly, we theorize that supervisors high in moral identity will regard UPB as more problematic and lose trust in the subordinate who engage in UPB. When moral identity is salient, supervisors care more about the unethical cues conveyed by UPB, even though it is intended to benefit the organization. For these supervisors, subordinates engaged in UPB lack of adherence to acceptable principles, thereby, are less trustworthy (Knoll and Gill, 2011; Tomlinson et al., 2020).

Moreover, a highly self-important moral identity will expand the “circle of moral regard” (Reed and Aquino, 2003). That is to say, supervisors high in moral identity will feel a stronger moral obligation to show concern for the needs and interests of a larger society, rather than focusing on the benefits of their organization. In this regard, UPB could be interpreted more negatively, as it violates the social norm and damages the out-group interests although it is beneficial to the in-group. As a result, for supervisors with high moral identity, subordinates engaged in UPB are less trustworthy. Taken together, we expect that supervisors high in moral identity present less trust in subordinates who engage in UPB.

#### The role of supervisor goal congruence with the group

Rooted in the person-organization fit literature, goal congruence with the group captures the fit between individuals’ personal goal and the organizational goal (Chatman, 1989; Kristof, 1996; Vancouver and Schmitt, 2010), which is closely related to individual’s organizational identity (Cable and Derue, 2002; Edwards and Cable, 2009). When supervisors feel their personal goals match the group’s goal and value, they are inclined to adopt an organizational identity, feeling a sense of oneness with the organization (Ashforth and Mael, 1989), thus being more attentive to the attainment of organizational goals and interests.

The social identity theory provides important insights into how supervisors’ goal congruence with the group influences the information processing and perceptions of subordinates’ behavior (Tajfel and Turner, 1986; Hogg et al., 1995). Specifically, a salient organizational identity can result in greater sensitivity to information relevant to the organization. In a similar vein, research has suggested that information processing is a goal-driven process (Wyer and Srull, 1986), in which, information that pertains to this goal is retrieved, while those irrelevant to the goal could be lost in this procedure. Following this line of reasoning, goal congruence with the group, for its relevance to organizational goal and organizational identity, renders supervisors more focused on the pro-organizational nature of UPB, while neglecting its unethical aspects. As a result, subordinate UPB could be justified by its group-serving intentions (Effelsberg et al., 2014; Chen et al., 2016) and be recognized as loyalty or altruism toward the group, which leads to more trust in the subordinate (Knoll and Gill, 2011; Tomlinson et al., 2020).

In sum, our theorizing suggests that when supervisors’ moral identity is high and goal congruence with the group is low, they are more focused on the moral problem of UPB and less concerned about its pro-organizational aspect, which both result in less trust in subordinates. While these two predictions are elaborated separately above, the social identity theory suggests that individuals may simultaneously maintain multiple identities (Brown, 2000). Therefore, we integrate these two contingencies to expect that supervisors who are simultaneously high on moral identity and low on goal congruence with the group tend to be most attentive to the dark side of UPB while focusing least on its bright side, and thus regarding UPB as a sign of unreliability. Thus, we propose:

*Hypothesis* 1. There is a three-way interaction effect among subordinates’ unethical pro-organizational behavior, supervisors’ moral identity, and supervisors’ goal congruence with the group on supervisors’ trust in subordinates. Specifically, subordinates’ unethical pro-organizational behavior has the strongest negative relationship with supervisors’ trust in subordinates when supervisors’ goal congruence with the group is low and moral identity is high.

### The downstream effect on abusive supervisor behavior

Thus far, we have theorized the conditional effect of UPB on supervisor trust in the subordinate through information processing. Furthermore, as social judgment resulting from the information processing procedure is supposed to guide following behavioral responses (Wyer and Srull, 1986), we further expect that reduced trust will elicit supervisor abusive behavior. Building on prior research, we conceptualize abusive supervisor behavior, which refers to supervisors’ nonphysical hostility towards the subordinate, as punishment to subordinates who engage in UPB (Tepper, 2000; Smallfield et al., 2020).

There are several reasons to believe that supervisors’ reduced trust in subordinates will lead to subsequent abusive behavior. Drawing on a moral exclusion perspective (Tepper et al., 2011), when subordinates are perceived to be less trustworthy, especially due to their unethical behavior, they are considered undeserving of moral treatment. These morally excluded subordinates, therefore, are more likely to become targets for “exclusionary practice,” such as abusive supervisor behavior (Tepper et al., 2011). Moreover, from a relational perspective, out-group members, compared to those in-groups, are more liable to be targets of aggression (Miller et al., 2003). In this way, subordinates who are less trusted by their supervisors, as a result of their possible psychological out-group status, may encounter more abusive supervisor behavior. Supporting this reasoning, previous research has identified psychological contract violations, which is closely related to low levels of trust, as a contributor to abusive supervision (Hoobler and Brass, 2006).

In tandem with our earlier theorizing, we prose that supervisors who are both high in moral identity and low in goal congruence with the group will tend to evaluate subordinate’ s UPB as violating moral standards, thus contributing to lower trust in the subordinate and, ultimately, abusive supervisor behavior. We formalize this conditional indirect effect as follows:

*Hypothesis* 2. Supervisors’ trust in subordinates mediates the three-way interaction among subordinates’ unethical pro-organizational behavior, supervisors’ goal congruence with the group, and supervisors’ moral identity on abusive supervisor behavior. Specifically, subordinates’ unethical pro-organizational behavior has the strongest positive indirect effect on abusive supervisor behavior through supervisors’ trust in subordinates when supervisors’ goal congruence with the group is low and moral identity is high.

## Method

### Sample and procedure

We collected multi-wave, multiple-source data from eight state-owned enterprises in Beijing, Hubei Province, Shandong Province, and Hainan Province in China from May to June 2020. These enterprises belonged to a range of industries, including agriculture, energy, and property management. All participants were white-collar workers who performed professional, managerial, or administrative work in offices. Groups were formed from different departments (i.e., each group consisted of the head of the department and his/her direct subordinates). Survey samples were formed through a non-probability sampling mode that combines convenience and snowball sampling. Data were collected using a questionnaire. In particular, we contacted the heads of eight enterprises. In turn, they contacted departmental leaders in their organizations, who notified all members of the departmental staff to join the survey. Based on an introduction to study purposes, values, and confidentiality principles and on the consent of participants, an online questionnaire link was sent to participants through WeChat during the study period.

We initially provided surveys to 340 subordinates and 79 supervisors. Data were collected in three waves. During the first wave of data collection (time 1), subordinates provided data on their unethical pro-organizational behavior, counterproductive work behavior, and in-role task performance, and supervisors provided data on their general goal congruence with the group. Approximately 1 month later (time 2), supervisors evaluated their levels of trust in each subordinate as well as provided data on their general moral identity. Finally, subordinates rated supervisors’ abusive behaviors 1 month later (time 3). After removing unmatched and missing responses and those who failed to pass attention check guidelines, the final sample comprised 220 subordinates working under 66 different supervisors. The supervisors and members were included in four age groups, namely, below 29 years (1.5 and 24.8%, respectively), 30–39 years (25 and 42.8%, respectively), 40–49 years (48.5 and 20.7%, respectively), and over 50 years (23.5 and 7.3%, respectively). Females were 23.9 and 37.8% for supervisors and members, respectively. The average tenure in the current organization was 15.83 years for supervisors (SD = 8.99) and 9.55 years for members (*SD* = 7.64). The average length of the leader-member relationship was 3.85 years (*SD* = 3.60).

### Measures

We translated the survey from English into Chinese using back-translation procedures (Brislin, 1986). All responses used a five-point Likert-type scale (1 = completely disagree; 5 = completely agree).

#### Unethical pro-organizational behavior

We utilized the 6-item measure developed by Umphress et al. (2010) to assess followers’ unethical pro-organizational behavior. Sample items include “If it would help my organization, I would misrepresent the truth to make my organization look good” and “If needed, I would conceal information from the public that could be damaging to my organization.” Cronbach’s alpha was 0.89.

#### Supervisors’ trust in followers

We adapted the 6-item measure from Schaubroeck et al. (2011). Sample items include “We would both feel a sense of loss if my follower was transferred and we could no longer work together” and “Given my follower’s track record, I see no reason to doubt his/her competence.” Cronbach’s alpha was 0.90.

#### Abusive supervisor behavior

Subordinates rated the extent to which their supervisors engaged in abusive behaviors using five items adapted by Peng et al. (2014) from Tepper’s (2000) original scale. Sample items include “My supervisor tells me my thoughts or feelings are stupid” and “My supervisor puts me down in front of others.” Cronbach’s alpha was 0.97.

#### Supervisor’s moral identity

Supervisors reported the extent to which they generally endorse a set of ethical characteristics (e.g., caring, fair, generous, friendly, and honest) using four items developed by Aquino and Reed (2002). Sample items include “It would make me feel good to be a person who has these characteristics,” and “These characteristics reflect how I see myself right now.” Cronbach’s alpha was 0.92.

#### Supervisor’s goal congruence with the group

We measured supervisors’ goal congruence with the group using 3 items adapted from Cable and Derue’s (2002) measure of goal congruence. Sample items include “Goals that I pursue in life are very similar to the goals that my organization pursues.” and “My personal goals match my organization’s goals.” Cronbach’s alpha was 0.96.

#### Control variables

We controlled for in-role task performance and counterproductive work behavior to verify that unethical pro-organizational behavior has unique effects. In-role task performance was measured using 6 items (α = 0.90; e.g., “I adequately complete assigned duties,” “I fulfill responsibilities specified in the job description”) developed by Williams and Anderson (1991). Counterproductive work behavior was measured using 5 items (α = 0.93; e.g., “I put little effort into their work,” “I intentionally worked slower than could”) developed by Bennett and Robinson (2000). In addition, we also controlled for supervisor age and gender, and subordinate age and gender. Prior research has shown that these demographic variables can be linked to unethical behaviors (Umphress et al., 2010) and to supervisor abusive behaviors (Zhang and Bednall, 2016). We also conducted all our analyses without any controls. The results are essentially the same in terms of patterns and levels of significance.

### Analytic strategy

Given that our model contains variables at the group level (i.e., supervisor’s moral identity and goal congruence with the group) and individual level (i.e., unethical pro-organizational behavior, in-role task performance, counterproductive work behavior, supervisors’ trust in each follower, and followers’ ratings of empowering and abusive supervision s), we employed multilevel path analysis in Mplus 7.11 (Muthén and Muthén, 2012). In line with the recommendation of Hofmann et al. (2000) and Enders and Tofighi (2007), we group-mean-centered level 1 predictors and grand-mean-centered level 2 predictors.

Because subordinates were nested within supervisors, we tested whether supervisors’ trust in subordinates and abusive supervision varied between supervisors. The analysis showed that the variance of supervisors’ trust in subordinates at the group level was significant (ICC (1) = 0.63, *p* < 0.001). We thus used random intercepts for supervisors’ trust in subordinates. However, the variance of abusive supervision at the group level was relatively small (ICC (1) = 0.06, *p* > 0.1). Therefore, abusive supervisor behavior was modeled as fixed slopes. As others have done (e.g., Wang et al., 2011), we also modeled control variables (e.g., positive affect and study day) with fixed slopes.

## Results

### Descriptive statistics and preliminary results

Descriptive statistics, correlations, and scale reliabilities are shown in Table 1. UPB was not correlated with supervisors’ trust in subordinates (*r* = −0.06, *n.s.*) and was not correlated with abusive supervision (*r* = −0.02, *n.s.*). Supervisor’s trust in subordinates was negatively correlated with abusive supervision (*r* = −0.18, *p* < 0.01). In terms of control variables, in-role task performance was significantly correlated with abusive supervision (*r* = −0.19, *p* < 0.01), counterproductive work behavior was significantly correlated with abusive supervision (*r* = 0.23, *p* < 0.01); we thus controlled for these variables (Becker, 2005).

**Table 1:** Descriptive statistics and intercorrelations of study variables.

Variable	*M*	*SD*	1	2	3	4	5	6	7
**Individual-level**									
Subordinate gender^a^	1.37	0.48	–						
Subordinate age^b^	3.07	0.96	−0.08	–					
Unethical pro-organizational behavior	1.84	0.70	−0.24^**^	−0.04	(0.89)				
In-role task performance	4.45	0.54	−0.03	0.03	−0.18^**^	(0.90)			
Counterproductive work behavior	1.34	0.53	−0.08	−0.04	0.40^**^	−0.42^**^	(0.93)		
Supervisor’s trust in subordinates	4.02	0.60	−0.08	−0.18^**^	−0.06	0.02	−0.09	(0.90)	
Abusive supervisor behavior	1.97	0.75	−0.06	0.19^**^	−0.02	−0.19^**^	0.23^**^	−0.18^**^	(0.97)
*Group-Level*									
Supervisor gender^a^	0.23	0.42	–						
Supervisor age^b^	3.95	0.74	−0.07	–					
Supervisor’s moral identity	4.18	0.78	0.18^**^	−0.30^**^	(0.96)				
Supervisor’s goal congruence with the group	4.21	0.56	−0.15^**^	−0.26^**^	0.24^**^	(0.92)			

Level 1 *N* = 220, Level 2 *N* = 66.

a Gender: 1 = male, 2 = female.

b Age: 1 = below 25 years, 2 = 26–30 years, 3 = 31–40 years, 4 = 41–50 years, 5 = 51–60 years, 6 = above 60 years.

**p < 0.01.

Multilevel confirmatory factor analysis of the 24 items showed acceptable fit to the five-factor hypothesized model: *χ*^2^ (129) = 447.19, *p* < 0.01, comparative fit index (CFI) = 0.93, standardized root mean square residual (SRMR) = 0.06 and root mean square error of approximation (RMSEA) = 0.08. This model had significantly better fit than plausible alternative models in which any two level-1 variables were combined (1356.69 ≤ Δχ^2^ ≤ 1447.68) and in which the two level-2 variables were combined (Δχ^2^ = 167.46), supporting the discriminant validity of our set of focal variables.

### Test of hypotheses

**Figure 2:**
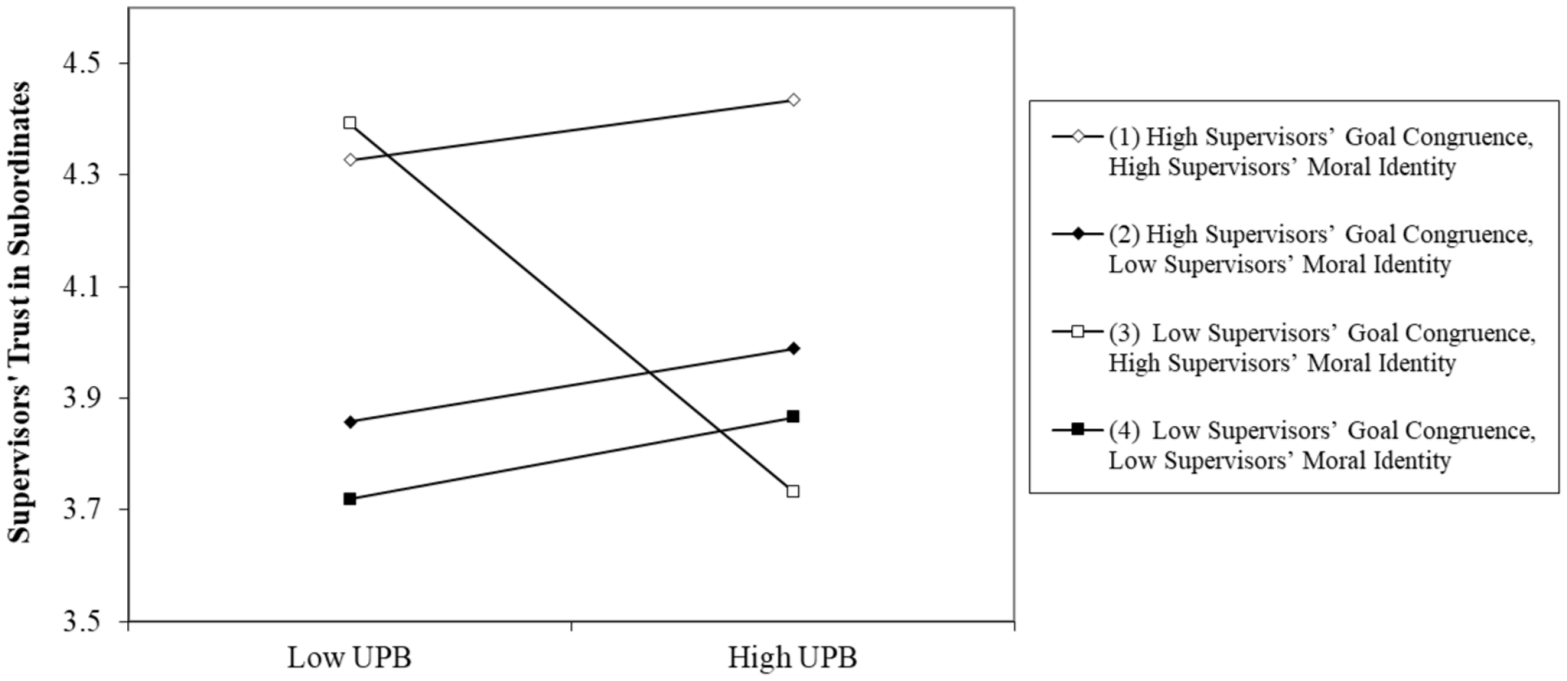
Three-way interaction among UPB, supervisors’ goal congruence, and supervisors’ moral identity.

The results regarding Hypothesis 1 are shown in Table 2. Hypothesis 1 predicted that UPB, supervisors’ goal congruence with the group, and supervisors’ moral identity interactively affect supervisors’ trust in subordinates such that UPB would have the strongest negative effect on supervisors’ trust in subordinates when supervisors’ goal congruence with the group is low and supervisors’ moral identity is high. The results indicated that the three-way interaction between UPB, supervisors’ goal congruence, and supervisors’ moral identity on supervisors’ trust in subordinates was significant (*B* = 0.44, *SE* = 0.21, *p* < 0.05). As shown in Figure 2 and Table 3, only when supervisors’ goal congruence with the group is low and supervisors’ moral identity is high, UPB had a significant, negative effect on supervisors’ trust in subordinates (*B* = −0.52, *SE* = 0.18, *p* < 0.01). In contrast, the effect of UPB on supervisors’ trust in subordinates was positive or nonsignificant for other combinations of supervisors’ goal congruence and supervisors’ moral identity. Specifically, the effect was significantly positive when supervisors’ goal congruence is high while supervisors’ moral identity is low (*B* = 0.21, *SE* = 0.09, *p* < 0.05), while it is nonsignificant when supervisor is both high or low on goal congruence with the group and moral identity (both high: *B* = 0.17, *SE* = 0.11, *n.s.*; both low: *B* = 0.19, *SE* = 0.12, *n.s.*). Taken together, Hypothesis 1 was supported.

**Table 2:** Multilevel modeling results for Hypothesis 1.

Predictors	Supervisors’ trust in subordinates			
	Model 1	Model 2	Model 3	Model 4
*Level 1*	–	–	–	–
Subordinate gender	0.12 (0.07)	0.12 (0.07)	0.10 (0.07)	0.12 (0.08)
Subordinate age	−0.03 (0.04)	−0.02 (0.04)	−0.02 (0.04)	−0.02 (0.04)
In-role task performance	0.06 (0.07)	0.06 (0.07)	0.06 (0.07)	0.06 (0.07)
Counterproductive work behavior	−0.08 (0.07)	−0.08 (0.07)	−0.09 (0.08)	−0.10 (0.07)
UPB	–	0.00 (0.05)	−0.01 (0.06)	−0.06 (0.06)
*Level 2*	–	–	–	–
Supervisor gender	−0.01 (0.14)	−0.03 (0.14)	−0.02 (0.14)	0.01 (0.14)
Supervisor age	−0.12 (0.08)	−0.01 (0.08)	0.01 (0.08)	−0.01 (0.08)
Supervisor’s moral identity (SMI)	–	0.13 (0.08)	0.15 (0.08)	0.15 (0.08)
Supervisor’s goal congruence (SGC)	–	0.36** (0.11)	0.35** (0.11)	0.35** (0.11)
*Interaction terms*	–	–	–	–
UPB * SMI	–	–	0.16* (0.07)	0.22** (0.08)
UPB * SGC	–	–	−0.22 (0.12)	−0.35** (0.13)
SMI * SGC	–	–	0.12 (0.14)	0.12 (0.14)
UPB * SMI * SGC	–	–	–	0.44* (0.21)

Level 1 *N* = 220, Level 2 *N* = 66. Unstandardized coefficients are presented and standard errors are shown in the parentheses. *p < 0.05, **p < 0.01.

**Table 3:** Conditional effects of UPB on creative self-efficacy.

Pairs of comparison	Slope	*SE*
1 (High SGC, high SMI)	0.17	0.11
2 (High SGC, low SMI)	0.21*	0.09
3 (Low SGC, High SMI)	−0.52**	0.18
4 (Low SGC, Low SMI)	0.19	0.12
*Slope difference*	–	–
(1) and (2)	−0.02	0.16
(1) and (3)	0.67**	0.25
(1) and (4)	−0.04	0.15
(2) and (3)	0.70**	0.21
(2) and (4)	−0.02	0.16
(3) and (4)	−0.72**	0.27

Level 1 *N* = 220, Level 2 *N* = 66. We computed the simple slopes with the values of the moderator(s) at one standard deviation above and below the mean. *p < 0.05, **p < 0.01.

Hypothesis 2 predicted that supervisors’ trust in subordinates mediates the conditional effect of UPB on abusive supervisor behavior such that UPB has the strongest positive, indirect effect when supervisors’ goal congruence with the group is low and supervisors’ moral identity is high. The results of multilevel modeling are shown in Table 4. As anticipated, supervisors’ trust in subordinates remained a significant, negative predictor of abusive supervision after accounting for control variables and UPB (*B* = −0.48, *SE* = 0.13, *p* < 0.01). The conditional indirect effect analysis using the Monte Carlo bootstrapping method (Preacher and Selig, 2012) showed that UPB had a positive, significant indirect effect on abusive supervisor behavior only when supervisors’ goal congruence with the group is low and supervisors’ moral identity is high (*B* = 0.08, *SE* = 0.05, 95% CI [0.07, 0.49]), but a negative, significant indirect effect when supervisors’ goal congruence with the group is high while supervisors’ moral identity is low (*B* = −0.07, *SE* = 0.04, 95% CI [−0.22, −0.01]). The indirect effect of UPB on abusive supervisor behavior was negative, but not significant when supervisors’ goal congruence with the group and supervisors’ moral identity were both low (*B* = −0.09, *SE* = 0.04, 95% CI [−0.23, 0.02]) or high (*B* = −0.08, *SE* = 0.04, 95% CI [−0.21, 0.02]). These results thus support Hypothesis 2.

**Table 4:** Multilevel modeling results for the moderated mediation model.

Predictors	Dependent variable	
	Supervisors’ trust in subordinates	Abusive supervisor behavior
*Level 1*	–	–
Subordinate gender	0.12 (0.08)	−0.04 (0.12)
Subordinate age	−0.02 (0.04)	0.07 (0.06)
In-role task performance	0.06 (0.07)	−0.12 (0.12)
Counterproductive work behavior	−0.10 (0.07)	0.26* (0.12)
UPB	−0.06 (0.06)	−0.10 (0.09)
*Level 2*	–	–
Supervisor gender	0.01 (0.14)	–
Supervisor age	−0.01 (0.08)	–
Supervisor’s moral identity (SMI)	0.15 (0.08)	–
Supervisor’s goal congruence (SGC)	0.37** (0.11)	–
*Interaction terms*	–	–
UPB * SMI	0.22** (0.08)	–
UPB * SGC	−0.36** (0.13)	–
SMI * SGC	0.12 (0.14)	–
UPB * SMI * SGC	0.45* (0.21)	–
*Mediator*	–	–
Supervisors’ trust in subordinates	–	−0.48** (0.13)
	Indirect effect	95% confidence interval
1 (High SGC, high SMI)	−0.08 (0.04)	[−0.21, 0.02]
2 (High SGC, low SMI)	0.07 (0.04)	[−0.22, −0.01]
3 (Low SGC, High SMI)	0.08 (0.05)	[0.07, 0.49]
4 (Low SGC, Low SMI)	−0.09 (0.04)	[−0.23, 0.02]

Level 1 *N* = 220, Level 2 *N* = 66. Unstandardized coefficients are presented and standard errors are shown in parentheses. *p < 0.05, **p < 0.01.

## Discussion

When subordinates go down the wrong path, supervisors are responsible for giving negative feedbacks to make them aware of their mistakes. However, supervisors may fail to fulfill this responsibility well in the face of subordinate UPB, which makes it important to understand when supervisors will respond negatively to subordinate UPB. In our study, we found that supervisors are most likely to lose trust in subordinates who engage in UPB when they are high in both moral identity and goal congruence with the group. Furthermore, the decrease of trust in the subordinate leads to abusive supervisor behavior.

### Theoretical implications

The current research made three primary contributions to existing literature. First, this paper advances the UPB literature by demonstrating the role of UPB in shaping supervisors’ attitudinal and behavioral reactions to subordinates. To date, current research has largely focused on antecedents of UPB (Chen et al., 2016; Wang et al., 2022), and less attention is paid to its consequences, especially how supervisors respond to subordinates’ UPB. While some researchers have begun to explore the consequences of UPB (e.g., Lian et al., 2020; Tang et al., 2020, 2021; Wang et al., 2022; Yang et al., 2022), their main focus is the influence of UPB on the actor or the trickle-down effect of supervisor UPB, and little is known about how supervisors react to subordinate UPB (for an exception, see Fehr et al., 2019). Understanding of this less studied question is of potential importance given that supervisors are responsible for providing proper feedback on subordinates’ behaviors, such that UPB, despite its short-term benefit, is supposed to be punished by the supervisors. In our paper, we suggest that supervisors are most likely to distrust those who engage in UPB and present abusive behaviors in response when they have both high goal congruence with the group and high moral identity. In this way, our study diverges from conventional approaches to UPB by exploring its critical consequences in terms of supervisors’ attitudinal and behavioral responses to it.

Second, we contribute to the abusive supervision literature by highlighting subordinates’ bottom-up influence on the supervisor in contrast to the often studied top-down effects. As leading behaviors exist in the dyadic interactions between the supervisor and the subordinate, understanding the upward impact of subordinates’ behavior is just as important as the downward impact of supervisors. Although there has been accumulative evidence on subordinate-related antecedents of abusive supervision (for reviews, see Zhang and Bednall, 2016; Fischer et al., 2021), they focus mainly on subordinates’ personalities or other static traits that induce abusive supervision (e.g., narcissism, neuroticism), ignoring the fact that supervisors can perform abusive behaviors due to some temporary behaviors of subordinates. In the current paper, specifically, we demonstrate that subordinates’ engaging in UPB will lead to abusive supervisor behavior, thus revealing the role of subordinate behaviors in the formation of abusive supervision. In addition, this result also offers the implication that abusive supervision can be targeted at specific behaviors rather than the person.

Third, we contribute to the moral identity literature by expanding scholars’ understanding of its impact. While moral identity has been widely studied as a predictor of individual’s own ethical behaviors (Jennings et al., 2014; Hertz and Krettenauer, 2016), we instead explore its impact on perceptions of others’ ethic related actions. Integrating social identity theory with social information processing theory, our findings suggest that moral identity affects how supervisors process information, rendering them more focused on the unethical nature of UPB and thus negatively response to it. This is in accordance with the arguments of social identity theory that identities provide individuals with available schemas for social information processing, which determines not only the way individuals behave but also the way they think and feel (Tajfel and Turner, 1986; Hogg et al., 1995). Overall, the present research enriches our understanding of the role that one’s moral identity plays in their cognition process.

### Practical implications

Our findings have several important implications for practice. First, given the potential positive short-term consequences of UPB, it is not surprising that supervisors may acquiesce in or even support subordinate UPB, while this is not wise indeed. As suggested by our results, supervisors who are low on goal congruence with the group and high on moral identity seem to be less likely to give in to such temptation and overlook the detriments of UPB. Given that goal congruence is to the advantage of individuals and the organization (De Clercq et al., 2014), the more important takeaway from our study for supervisors should be to develop their ethical awareness overall, rather than to avoid lining up with the group. Supervisors are highly suggested to focus on the big picture of the organization and pay particular attention to ethical issues in order to make proper decisions on UPB. Moreover, developing ethical climates within the organization brings many benefits (Martin and Cullen, 2006), helping both supervisors and subordinates to be aware of the ethical principles when making decisions.

Second, our findings also warrant caution of supervisors to avoid “overreacting” to employees’ UPB. According to our results, supervisors may mistreat the subordinate who engages in UPB. Despite the fact that UPB should be treated in a negative way, abusive supervisor behaviors in response still cannot be justified as a way of punishment or expression of negative attitude. In fact, supervisors may hold that such behavior is an instrumental attempt to punish misbehaviors and stimulate performance promotions (Ferris et al., 2007; Tepper, 2007; Krasikova et al., 2013), yet research has debunked this argument (Walter et al., 2015), and abusive supervision has long been proved detrimental to both subordinates and the organization (Tepper, 2007; Martinko et al., 2013). Therefore, even supervisors who are aware of the darkside of UPB should pay additional attention to restrain their potential abusive behaviors in response, and find more effective ways to remind subordinates of their unwanted behaviors.

Third, our study further provides implications for employees who are suffering from abusive supervision. Previous research has established that abusive supervision is likely to be perceived as personal aggression and injustice, which ultimately translates into negative emotions (e.g., anger, fear) and deviant behaviors (Tepper, 2000; Oh and Farh, 2017). However, as suggested by our results, abusive supervisor behaviors can be a result of subordinates’ inappropriate conduct rather than their personality or traits. In light of this, subordinates are suggested not to take abusive supervision personally, and if reflection is needed, it should be “what is wrong with things that I am doing” instead of “what is wrong with me?”

### Limitations and directions for future research

Despite the strengths of our studies, there are several limitations, from some of which opportunities for future research are highlighted. First, although we focus on supervisors’ behavioral responses to UPB, we have subordinates report their supervisor’s abusive supervision. This approach assumes that there is an agreement between subordinates’ perception of abusive supervision and actual abusive behaviors that supervisors conduct, while subordinates may underreport their being victimized or exaggerate their supervisors’ abusive supervision (Tepper et al., 2006). However, given that a self-reported abusive supervision by the supervisor could raise even higher concerns about the social desirability bias, our measurement should be reasonable. In addition, considering common method variance, it is better to have the dependent variable (i.e., abusive supervisor behavior) reported by subordinates because the mediator (i.e., supervisor’s trust in subordinates) is more suitable for self-reporting. For a similar reason, UPB is reported by subordinates. This may raise concerns about the inconsistency between our theoretical arguments and the measurement, given that the influence of UPB on supervisor trust and abusive behavior is based on supervisors’ perception of rather than the actual subordinate UPB. Therefore, we recommend future studies to collect data from different sources to measure abusive supervision and subordinate UPB.

Second, although abusive supervisor behavior is framed as a punishment for UPB in our work, we admit that as a typical destructive leading behavior, abusive supervision should in no way be supported. While supervisors may justify their abusive behaviors as an instrumental attempt to stimulate better performance (Ferris et al., 2007; Tepper, 2007; Krasikova et al., 2013), research has refuted this argument (Walter et al., 2015). Consequently, an important direction for future research is to explore other punitive behavioral responses of supervisors to subordinate UPB so as to see when supervisors will respond to UPB correctly but not excessively. Moreover, scholars may further consider whether punitive supervisor behaviors can actually reduce subordinate UPB as intended.

Third, although we included in-role task performance and counterproductive work behavior as control variables, we are unable to control for all variables that may be related to supervisor trust and abusive behaviors. In addition, the relationship between subordinate UPB and abusive supervision might be contingent on factors beyond supervisor characteristics. For example, whether supervisors choose to punish subordinate UPB or not might also depend on their relationships with the subordinate or other subordinate-related factors. Therefore, we encourage future research to explore these possibilities in depth and offer a more comprehensive understanding of supervisors’ responses to subordinate UPB.

## Data availability statement

The raw data supporting the conclusions of this article will be made available by the authors, without undue reservation.

## Ethics statement

Ethical review and approval was not required for the study on human participants in accordance with the local legislation and institutional requirements. The patients/participants provided their written informed consent to participate in this study.

## Author contributions

FG, YW, and JZ contributed to the conception and design of the study, and wrote sections of the manuscript. YW organized the database and performed the statistical analysis. FG wrote the first draft of the manuscript. All authors contributed to the manuscript revision, read, and approved the submitted version.

## Funding

This study was funded by the National Natural Science Foundation of China (No: 72102228) and the Outstanding Innovative Talents Cultivation Funded Programs 2021 of Renmin University of China.

## Conflict of interest

The authors declare that the research was conducted in the absence of any commercial or financial relationships that could be construed as a potential conflict of interest.

## Publisher’s note

All claims expressed in this article are solely those of the authors and do not necessarily represent those of their affiliated organizations, or those of the publisher, the editors and the reviewers. Any product that may be evaluated in this article, or claim that may be made by its manufacturer, is not guaranteed or endorsed by the publisher.
